# Supplementary material for: An Embryonic Zebrafish Model to Screen Disruption of Gut-Vascular Barrier upon Exposure to Ambient Ultrafine Particles
Source: Toxics. 2020 Nov 19;8(4):107. doi: 10.3390/toxics8040107 (PMC7711522; doi:10.3390/toxics8040107)
Supplement: Supplementary file 1 [file toxics-08-00107-s001.pdf]

toxics

Supplementary Materials: An Embryonic Zebrafish Model to Screen Disruption of Gut-Vascular Barriers upon Exposure to Ambient Ultrafine Particles

Kyung In Baek, Yi Qian, Chih-Chiang Chang, Ryan O'Donnell, Ehsan Soleimanian, Constantinos Sioutas, Rongsong Li and Tzung K. Hsiai

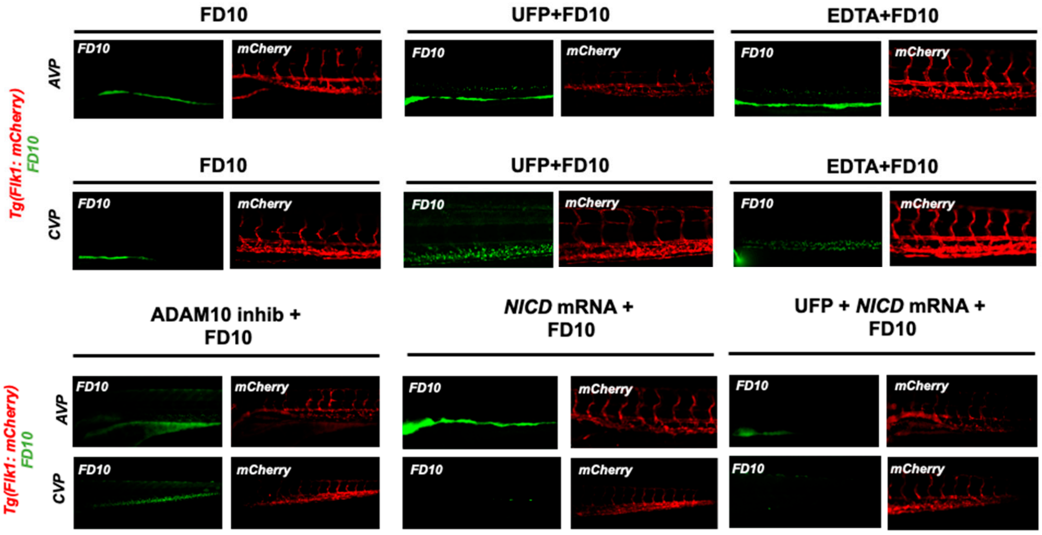

Figure S1. Imaging endoluminal distribution of FD10. Images of FD10 (green) and vascular endothelium (*flk1*$^{+}$, red) were taken respectively in response to UFP, Adam10 inhibitor, and *NICD* mRNA rescue and superimposed to assess vascular endothelial distribution of FD10.
